# Large dissecting sinus of Valsalva aneurysm creating a ‘triple ventricle’ appearance: case report

**DOI:** 10.1093/ehjcr/ytaf308

**Published:** 2025-07-15

**Authors:** Swasthi S Kumar, Sudipta Mondal, Jayakrishnan Radhakrishnan, Ramya Das

**Affiliations:** Department of Cardiology, Sree Chitra Tirunal Institute for Medical Sciences and Technology, Thiruvananthapuram, Kerala 695011, India; Department of Cardiology, Sree Chitra Tirunal Institute for Medical Sciences and Technology, Thiruvananthapuram, Kerala 695011, India; Department of Imaging Sciences and Interventional Radiology, Sree Chitra Tirunal Institute for Medical Sciences and Technology, Thiruvananthapuram, Kerala 695011, India; Department of Cardiology, Sree Chitra Tirunal Institute for Medical Sciences and Technology, Thiruvananthapuram, Kerala 695011, India

**Keywords:** Sinus of Valsalva aneurysm, Dissection of ventricular septum, Dissecting aneurysm, Case report

## Abstract

**Background:**

Dissecting aneurysm of the interventricular septum (DAIS) is a rare congenital or acquired anomaly which can have a progressive course. We report a large DAIS incidentally detected by a routine echocardiogram.

**Case summary:**

An asymptomatic tricenarian was incidentally detected to a DAIS during a routine pre-operative evaluation. Multimodality imaging with cardiac CT, MRI, and aortic root angiograms confirmed the diagnosis. Work-up for infectious causes like syphilis and inflammatory causes like connective tissue disorders were negative. Patient is planned for surgical repair.

**Discussion:**

Dissecting aneurysm of the interventricular septum is a rare anomaly with a poor prognosis. The rupture of sinus of Valsalva aneurysm is the most common cause. Infections like syphilis and infective endocarditis, connective tissue disorders (Marfan syndrome and Ehler–Danlos syndrome), autoimmune diseases (Behcet’s disease, ankylosing spondylitis, systemic lupus erythematosus, Takayasu’s arteritis), bicuspid aortic valve with aortopathy, atherosclerosis, surgery, or trauma are the reported causes. Such dissecting aneurysms can have a progressive course, leading to right or left ventricular outflow tract obstructions, severe aortic regurgitation, and rupture into ventricular chambers, portending a grave prognosis. Hence, surgical correction is the norm.

Learning points

- Dissecting sinus of Valsalva aneurysms is rare congenital or acquired anomalies which have a progressive course leading to rupture, right or left ventricular outflow tract obstruction, aortic regurgitation, and conduction abnormalities.
- Multimodality imaging with echo, CT, MRI, and aortic angiogram helps in delineating the aneurysm.
- Surgical correction is the norm.

## Introduction

Dissecting aneurysm of the interventricular septum (DAIS) is a rare anomaly, which may be congenital or acquired as a complication of sinus of Valsalva aneurysm (SOVA), surgery, or infective endocarditis.^1^ Such dissecting aneurysms can have a progressive course, leading to right or left ventricular outflow tract obstructions, severe aortic regurgitation (AR), and rupture into ventricular chambers, portending a grave prognosis.^2^ Hence, urgent surgical correction is the norm. We report a large DAIS incidentally detected by a routine echocardiogram.^1,3^

## Summary figure

**Figure:**
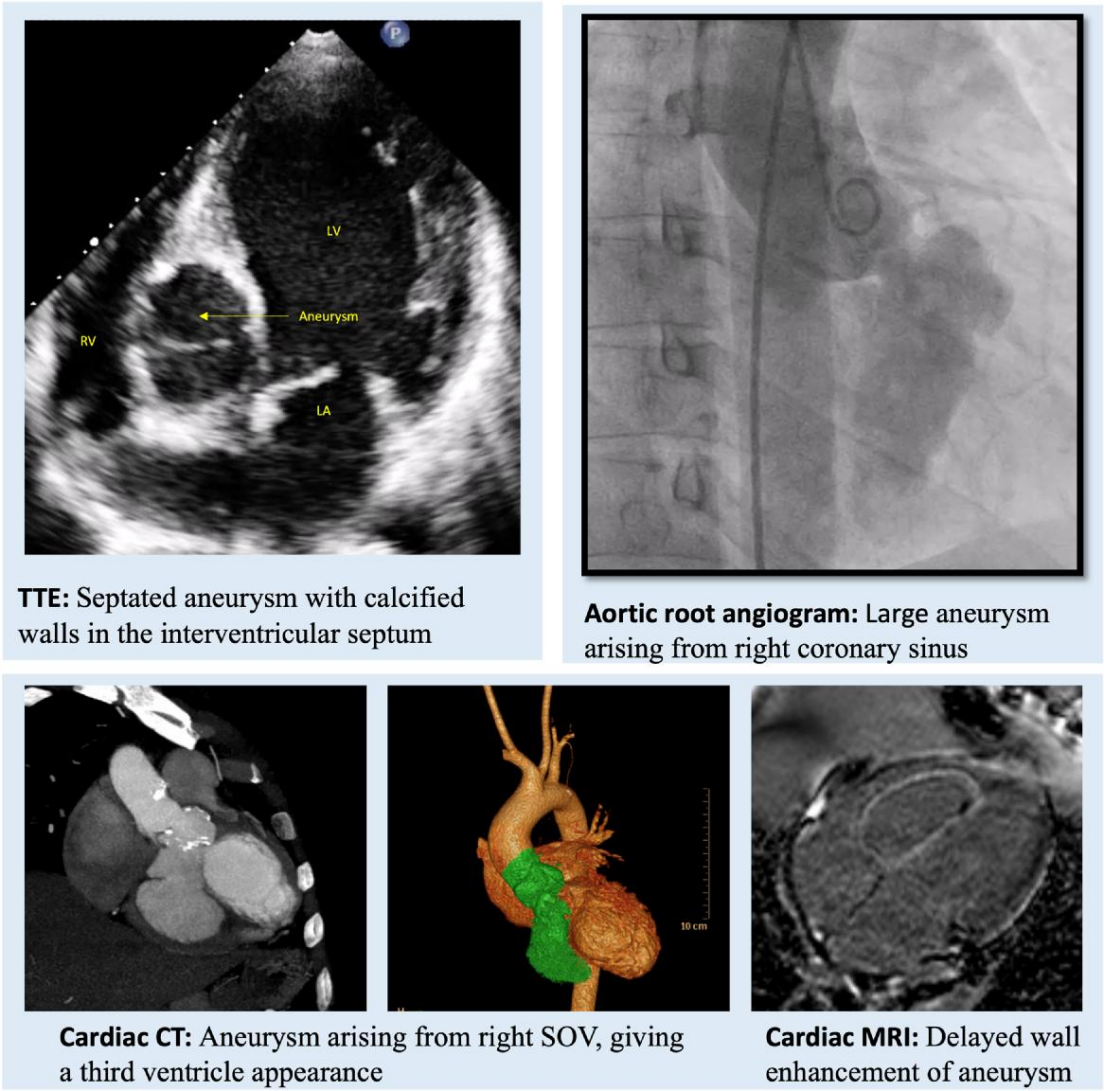


## Case presentation

**Figure 1:**
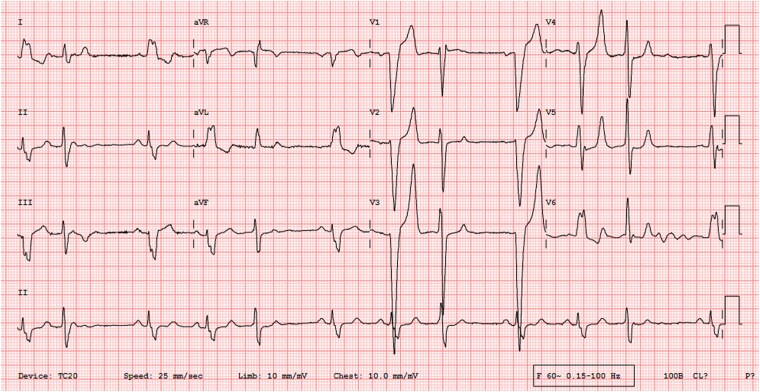
Twelve-lead electrocardiogram showing sinus rhythm with normal PR interval, left bundle branch block (LBBB), and frequent premature ventricular complexes (PVCs) of right bundle branch block morphology, superior and left axis, and late transition suggesting a posterior-basal septal origin.

**Figure 2:**
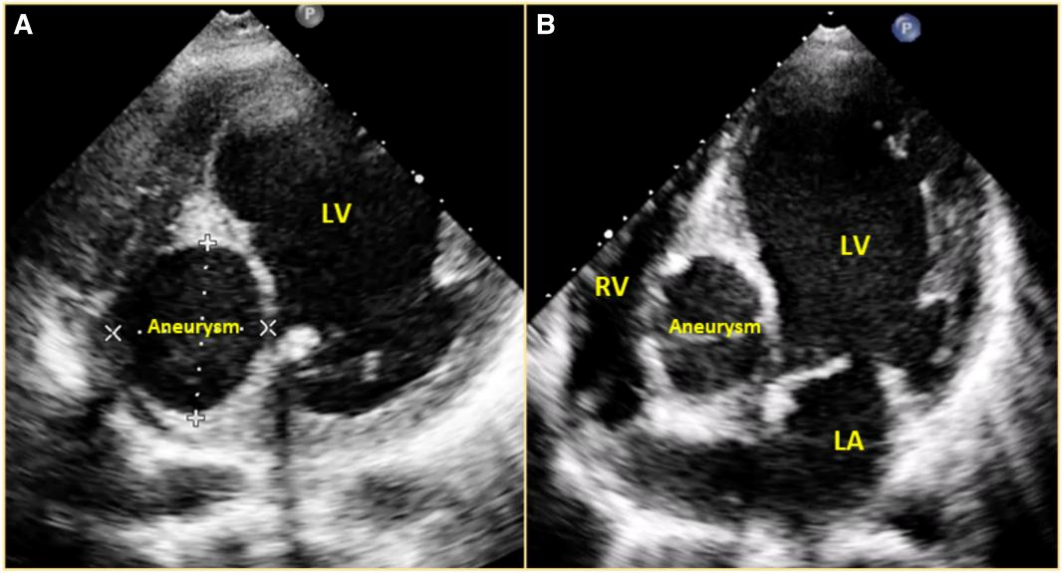
2D echocardiogram in modified short axis view in midventricular segment (*A*) and modified apical four-chamber view (*B*) showing 37 mm × 42 mm aneurysm dissecting into the interventricular septum with calcified walls. LV, left ventricle; RV, right ventricle; LA, left atrium.

An asymptomatic man in his thirties, without any known comorbidities, underwent an echocardiogram as part of a pre-operative evaluation for an inguinal hernia after the identification of an abnormal clinical examination and electrocardiogram. Clinical examination revealed normal room air oxygen saturation, jugular venous pressure, and blood pressure. Pulse was irregularly irregular. Cardiovascular assessment identified mild cardiomegaly with a 2/6 pansystolic murmur at the cardiac apex and a left ventricular S3. The electrocardiogram revealed sinus rhythm with normal PR interval, left bundle branch block (LBBB), and frequent premature ventricular complexes (PVCs) of right bundle branch block morphology, superior and left axis, suggesting a posterior-basal septal origin (*Figure 1*). The echocardiogram showed severe left ventricular (LV) dysfunction (LV ejection fraction 30%) with moderate mitral regurgitation (MR) (effective regurgitant orifice area 0.26 cm^2^, vena contracta 0.4 cm, regurgitant volume 42 mL, and radius for proximal isovelocity surface area was 7 mm). A 37 mm × 42 mm aneurysm was seen arising from the right coronary sinus, dissecting into the interventricular septum (IVS) (*Figure 2A, B*). Mild aortic regurgitation was observed in the presence of a tricuspid aortic valve. No communication was seen between the aneurysm sac and the ventricular cavity.

**Figure 3:**
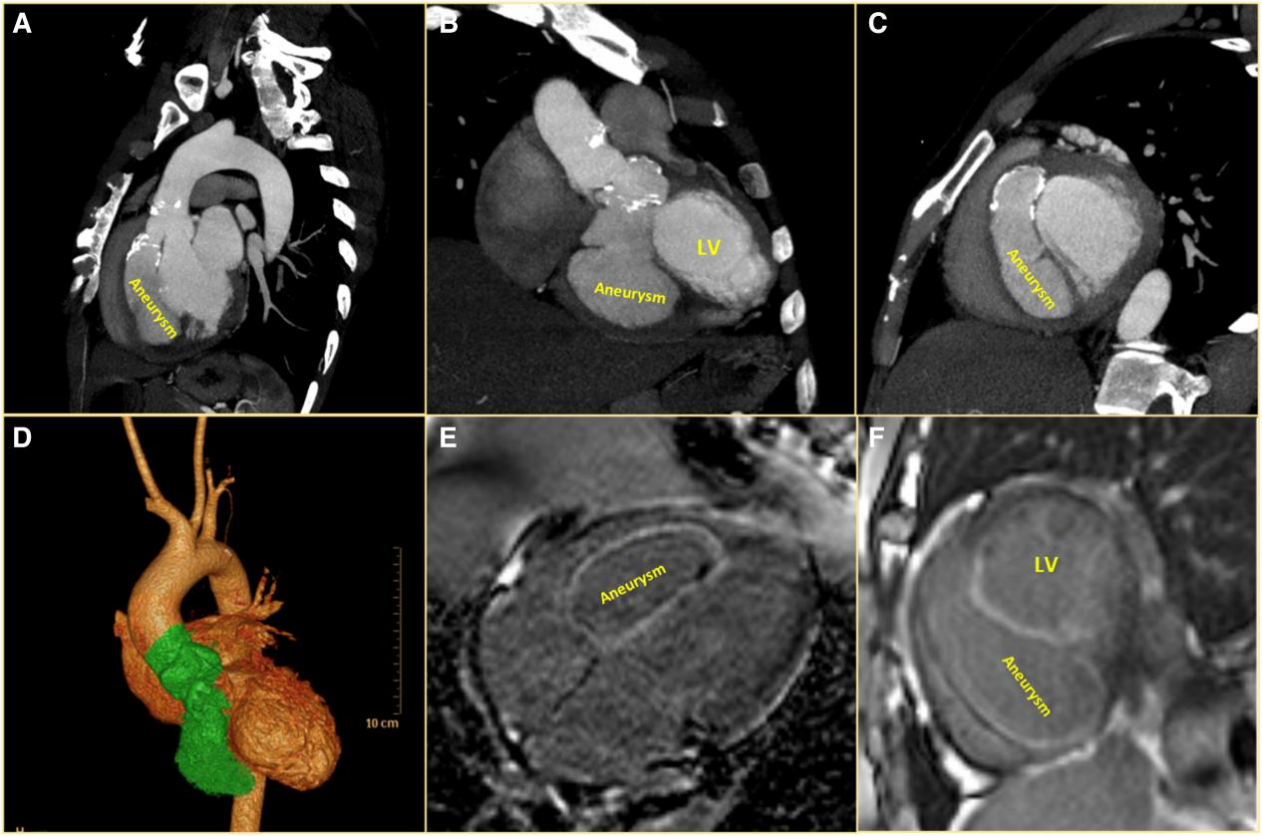
Sagittal (*A*) and coronal (*B*) cardiac computed tomography (CT) images showing the aneurysm arising from the right sinus of Valsalva dissecting into the interventricular septum. Short axis reconstructed CT image (*C*) showing the ‘triple ventricle’ appearance formed by the large dissecting aneurysm. (*D*) CT volume rendered image highlighting the sinus of Valsalva aneurysm in green (shaded in grayscale version). Horizontal long axis (*E*) and short axis (*F*) phase-sensitive inversion recovery MRI images showing the same aneurysm. LV, left ventricle.

Cardiac computed tomography (CT) (*Figure 3A–D*) confirmed a contrast-filled saccular blind-ending outpouching arising from the right coronary sinus measuring 81 mm × 37 mm via a rent measuring 14 mm, located at 16 mm from RCA origin. The outpouching was found to be dissecting a plane along the IVS up to the mid-ventricular level. There was resultant thinning of IVS with a dyskinetic motion of anteroseptal, inferoseptal basal, and mid-ventricular segments. Multifocal calcifications were seen in the neck of the outpouching. Outpouching was not communicating with the LV cavity. CT aortogram also showed narrowing at the origin of the coeliac trunk, with a thrombosed superior mesenteric (collateralized from an internal mammary artery) and left renal artery.

Cardiac MRI (*Figure 3E, F*) showed a perfusion defect and late gadolinium enhancement in the ventricular septum along the walls of the aneurysm. A thin rim of non-enhancing thrombus was noted in the anterior wall of the aneurysm sac at the mid-ventricular level. The right ventricular (RV) cavity was compromised by the aneurysm bulging into the RV.

The patient was extensively worked up for inflammatory and infectious aetiologies. Procalcitonin, blood culture, and inflammatory markers were negative. The antinuclear antibody was weakly positive at a dilution of 1:320. Antiphospholipid and anti-neutrophilic cytoplasmic antibodies were negative. The adenosine deaminase-2 gene showed no pathological variation. HLA-B21 (human leucocyte antigen—B21), the venereal disease research laboratory and rapid plasma reagin were negative. Though there was involvement of renal and mesenteric arteries (likely consequent to embolization), the diagnostic criteria for Takayasu’s arteritis were not fulfilled. Genetic testing (whole-exome sequencing) revealed no pathogenic mutation.

**Figure 4:**
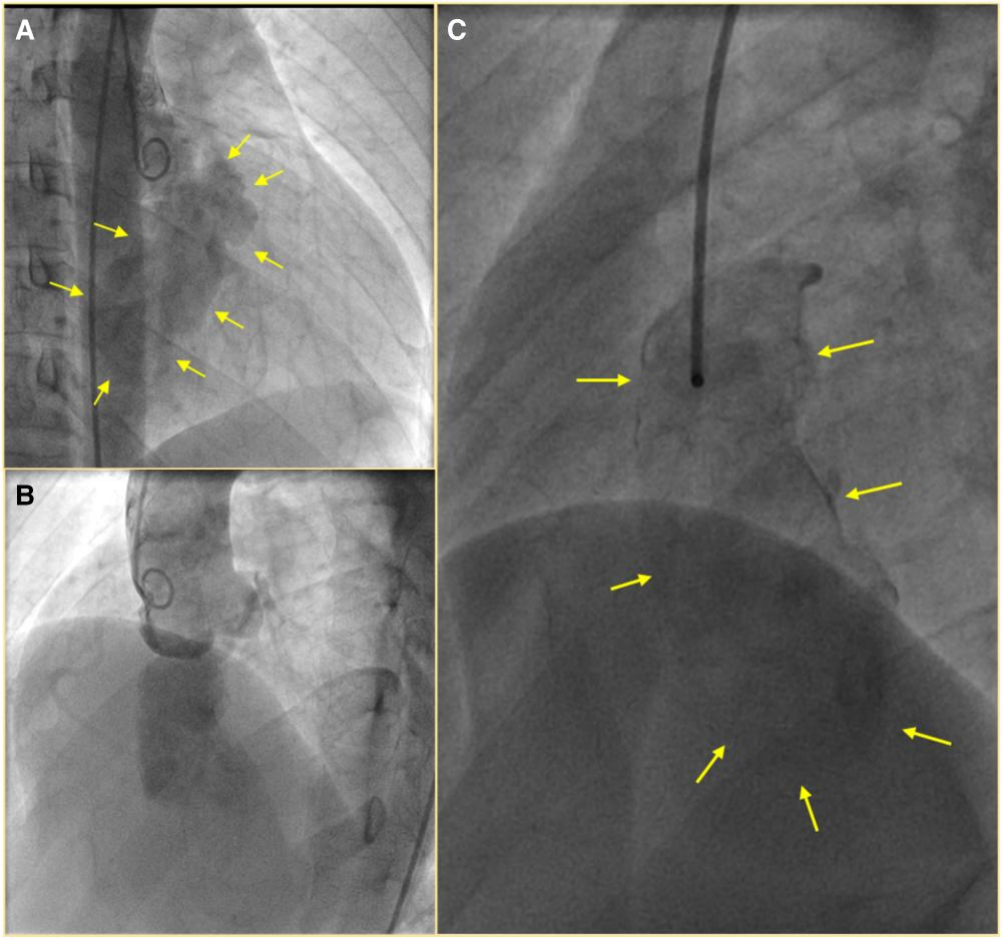
Aortic root angiogram in right anterior oblique view (*A*) and left anterior oblique—cranial view (*B*) showing a large aneurysm with calcified walls arising from the right coronary sinus extending into the ventricular septum. (*C*) Selective angiogram of the aneurysm clearly demarcating (arrows) the extension of the aneurysm into the ventricular wall. Note that there was no communication with any of the ventricular cavities.

A coronary angiogram showed no flow-limiting lesions in the coronaries. The aortic root angiogram showed a large aneurysm with calcified walls arising from the right coronary sinus and extending into the ventricular septum (*Figure 4A–C*). Contrast stasis was noted in the aneurysm sac. Although urgent surgical intervention was recommended, the patient declined the procedure for personal reasons and was subsequently lost to follow-up.

## Discussion

Dissecting aneurysm of the interventricular septum, first documented by Warthen in 1947, is histopathologically characterized as a *pseudoaneurysm* rather than a true aneurysm, as it lacks a complete true arterial wall.^4,5^ These aneurysms are more prevalent in men of Asian heritage. The most common precursor to DAIS is a *SOVA*.^6^ Sinus of Valsalva aneurysms develops due to thinning of the aortic sinus walls, primarily from a deficiency of elastic tissue in the tunica media. Prolonged exposure to high pressure then causes the sinus to become aneurysmal. Various factors contribute to the development of SOVAs, including infections like syphilis and infective endocarditis, connective tissue disorders (e.g. Marfan syndrome and Ehlers–Danlos syndrome), autoimmune diseases (e.g. Behcet’s disease, ankylosing spondylitis, systemic lupus erythematosus, and Takayasu’s arteritis), bicuspid aortic valve with aortopathy, atherosclerosis, and even prior surgery or trauma. The majority (75%) of SOVAs originate from the right coronary cusp, followed by the non-coronary cusp at 23%, and rarely from the left coronary cusp.^7^ Sinus of Valsalva aneurysms commonly ruptures into the RV, followed by the right atrium (RA). Less frequent rupture sites include the left ventricle (LV) and pulmonary artery. Importantly, a SOVA can also dissect into the IVS without directly communicating with a cardiac chamber, which can potentially lead to DAIS.

Dissecting aneurysm of the interventricular septum is a rare anomaly associated with a *poor prognosis* due to its progressive nature and risk of gradual expansion with subsequent communication to a cardiac chamber. Existing literature highlights various presentations: congestive heart failure (54%), palpitations (31%), syncope (27%), chest pain (15%), sudden cardiac death (4%), infective endocarditis (4%), stroke (4%), and asymptomatic occurrence (4%).^8^

Our patient, for instance, presented with *asymptomatic severe LV dysfunction*. This was likely caused by a calcific aneurysmal, dyskinetic septum with limited contractile contribution, potentially exacerbated by moderate MR, LBBB, and frequent PVCs. While mild AR can result from aortic cusp or root distortion, *severe AR should prompt suspicion of aneurysm rupture into the LV or aortic valve endocarditis*. Furthermore, the pressure effect or low-grade inflammation of conduction tissue can lead to various conduction disturbances, from first-degree atrioventricular block to complete heart block.^8^ Intriguingly, our patient exhibited LBBB with probable PVCs originating from the calcified aneurysmal septal wall. This finding suggests the proximal septal extension of the calcific aneurysm, potentially acting as a source of ventricular ectopy. Differential diagnoses for such an aneurysmal sac in the IVS include hydatidosis, IVS abscess, or a septal tricuspid leaflet aneurysm prolapsing into the LV through a partially closed ventricular septal defect. Given the absence of an acquired aetiology, a congenital cause was considered highly probable in this specific case.^1,8^

*Urgent surgical intervention is the standard of care for DAIS* given the significant risk of progression to rupture and subsequent haemodynamic deterioration. Some centres advocate for *conservative surgical approaches*, which involve directly closing the aneurysm’s mouth or patching it, without attempting to resect the entire aneurysm sac or obliterate the cavity. In such cases, the absence of distending aortic pressure allows the aneurysm cavity to thrombose and appear slit-like on follow-up imaging.^8^ Complete resection of a SOVA is rarely feasible due to the risk of damaging surrounding vital structures. Any exploration of the ventricular septum carries inherent hazards, and iatrogenic ventricular septal defect must be meticulously avoided by any methods.

Despite the necessity of intervention, the perioperative mortality rate for DAIS stands at 3.9%. Patients also face potential post-surgical complications, including left ventricular outflow tract obstruction, life-threatening tachyarrhythmias, complete heart block, and intractable heart failure if communication with the LV persists.^8^

## Conclusion

Dissecting aneurysm of the interventricular septum can be completely asymptomatic and diagnosed incidentally. Urgent surgical management is the standard given the risk of progression to rupture or to other serious mechanical complications. Multimodality imaging assessment and meticulous surgical planning are of the essence, as well as careful discussion with the patient and relatives.

## Data Availability

All data are incorporated into the article.

## Lead author biography

Senior resident at the Department of Cardiology of Sree Chitra Tirunal Institute of Medical Sciences and Technology.

## Notes

**Consent:** The authors confirm that written consent for submission and publication of this case report, including images and associated text, has been obtained from the patient in line with COPE guidance.

**Funding:** None.

**Ethics approval:** The study was conducted in accordance with the ethical standards laid down in the 1964 Declaration of Helsinki and its amendments. Informed written consent was taken from the patient concerned. No patient identity particulars have been disclosed.
